# siRNA nanoparticle targeting *Usp20* lowers lipid levels and ameliorates metabolic syndrome in mice

**DOI:** 10.1016/j.jlr.2024.100626

**Published:** 2024-08-22

**Authors:** Yi Ding, Qiu-Bing Chen, Hui Xu, Dilare Adi, Yi-Wen Ding, Wen-Jun Luo, Wen-Zhuo Zhu, Jia-Chen Xu, Xiaolu Zhao, Xiong-Jie Shi, Jie Luo, Hao Yin, Xiao-Yi Lu

**Affiliations:** 1 College of Life Sciences, Hubei Key Laboratory of Cell Homeostasis, Taikang Center for Life and Medical Sciences, Taikang Medical School, Wuhan University, Wuhan, China; 2 Department of Urology, Frontier Science Center for Immunology and Metabolism Medical Research Institute, Zhongnan Hospital of Wuhan University, Wuhan University, Wuhan, China; 3 Heart Center, First Affiliated Hospital of Xinjiang Medical University, Urumqi, Xinjiang, China

**Keywords:** cholesterol, lipid nanoparticles, HMGCR, USP20, atherosclerosis

## Abstract

Atherosclerotic cardiovascular disease is closely correlated with elevated low density lipoprotein-cholesterol. In feeding state, glucose and insulin activate mammalian target of rapamycin 1 that phosphorylates the deubiquitylase ubiquitin-specific peptidase 20 (USP20). USP20 then stabilizes HMG-CoA reductase, thereby increasing lipid biosynthesis. In this study, we applied clinically approved lipid nanoparticles to encapsulate the siRNA targeting *Usp20*. We demonstrated that silencing of hepatic *Usp20* by siRNA decreased body weight, improved insulin sensitivity, and increased energy expenditure through elevating UCP1. In *Ldlr*^−/−^ mice, silencing *Usp20* by siRNA decreased lipid levels and prevented atherosclerosis. This study suggests that the RNAi-based therapy targeting hepatic *Usp20* has a translational potential to treat metabolic disease.

A high level of low density lipoprotein (LDL)-cholesterol is a key risk factor for the atherosclerotic cardiovascular disease (1, 2). The cholesterol-lowering drugs such as the HMG-CoA reductase (HMGCR) inhibitor statins, the cholesterol absorption inhibitor ezetimibe, and the proprotein convertase subtilisin/kexin 9 inhibitors are widely used to treat atherosclerotic cardiovascular disease (3, 4).

In the cholesterol biosynthetic pathway, HMGCR is the rate-limiting enzyme that converts HMG-CoA to mevalonate (5, 6). Mammalian cells regulate cholesterol biosynthesis at both mRNA and protein levels. High levels of cholesterol suppress transcription of the genes encoding cholesterol biosynthetic enzymes. Meanwhile, high levels of sterols, such as lanosterol and oxysterols, stimulate HMGCR degradation by promoting its binding to Insig and the associated ubiquitin ligases E3s (7, 8, 9, 10), which ubiquitinate HMGCR and accelerate HMGCR turnover. Gp78 is the major E3 for HMGCR degradation in the liver that is the central organ for lipid metabolism (10, 11). These feedback regulatory mechanisms prevent mammalian cells from making too much cholesterol.

Besides negative feedback regulation, cholesterol biosynthesis is dramatically increased after eating carbohydrate-rich foods. The postprandial increase in insulin and glucose activates mammalian target of rapamycin 1 to phosphorylate the deubiquitylase ubiquitin-specific peptidase 20 (USP20). USP20 then binds gp78 and antagonizes the degradation of HMGCR. By this mechanism, HMGCR protein level is increased by about 20-fold, and cholesterol biosynthetic rate is increased by about 10-fold in the feeding state compared with the fasting state (12). Genetic deletion or pharmacological inhibition of USP20 reduces body weight, increases energy expenditure, and improves glucose clearance. Hence, USP20 is a promising target for treatment of metabolic syndrome (12, 13).

The nucleic acid-based therapeutic methods have been widely used to treat a variety of diseases (14, 15, 16, 17, 18). The short-interfering RNA (siRNA) is composed of double-stranded RNA molecules that are usually 21–23 nucleotides in length and have the ability to silence the expression of specific genes (19, 20). To improve siRNA stability and targeting affinity, chemical modifications and advanced delivery technologies such as lipid nanoparticle (LNP) systems have been developed (21). LNPs are the most clinically advanced nonviral vectors and have gained widespread acceptance as a result of the successful use of mRNA vaccines developed by Moderna and Pfizer/BioNTech and siRNA therapeutics by Alnylam (22, 23, 24). LNPs encapsulate siRNA molecules in a lipid-based nanoparticle to protect the siRNA from degradation in the bloodstream while facilitating its uptake by target cells (25, 26). The siRNA–LNP complex can be designed to be highly specific for liver or can be engineered to target tissues or cells beyond hepatocytes (27, 28). For instance, the first liver-targeting siRNA medicine was created by wrapping siRNA in MC3-siRNA targeting transthyretin (TTR) LNPs made up of four components: Dlin-MC3-DMA, cholesterol, DSPC, and PEG2000-DMG as carriers (29). This drug was approved by the US Food and Drug Administration in 2018 for the treatment of hereditary amyloidogenic transthyretin amyloidosis (30). The LNP technology is used to deliver TTR siRNA into hepatocytes cells, preventing mutant TTR protein production and subsequent fibril formation (31).

To prove the concept that knockdown of *Usp20* can treat metabolic disease, we used LNP-encapsulated siRNA to silence hepatic *Usp20* in mice and analyzed the metabolic profile. The results showed that silencing *Usp20* by LNP-siRNA reduced lipid contents in serum, decreased body weight, and prevented atherosclerosis.

## Materials and Methods

### Preparation of LNPs containing siRNA

D-Lin-MC3-DMA, DSPC, and cholesterol were obtained from AVT Pharmaceutical Tech Co., Ltd (Shanghai, China). C14-PEG2000 was purchased from Avanti Polar Lipids (Alabaster, AL). Quant-it™ RiboGreen RNA Assay Kit was purchased from Thermo Fisher Scientific (Waltham, MA). Eight siRNA target mouse *Usp20* sequences with the lowest off-target potential were bioinformatically selected as previously described (1). siRNAs were synthesized at Genescript (Nanjing, China). The certain sites of these siRNAs were introduced specific chemical modifications, including 2′-O-methylation and 3′ dTsdT. For the siRNAs screen, siRNA was transfected into Hepa1-6 cells and primary hepatocyte, respectively, using lipofectamine™ RNAiMax transfection reagent. Total RNA and protein were extracted for quantitative PCR (qPCR) and Western blot assay after transfecting for 24 h. LNPs were formulated as previously described (19, 20). Briefly, D-Lin-MC3-DMA, DSPC, cholesterol, and C14-PEG2000, with a molar ratio of ∼50/10/38.5/1.5, were dissolved in ethanol solution, and the siRNA was diluted in the 10 mM citrate buffer (pH = 4). Then the lipid solution and siRNA solution were injected into the microfluidic device at a relative volumetric flow rate of 1:3 (total flow rate: 12 ml/min). Finally, the freshly prepared LNPs were dialyzed for 2 h at room temperature and then stored in 4°C for further use. The siRNA encapsulation was determined by RiboGreen assay.

### Animals

Male C57BL/6J mice (6–8 weeks of age) were purchased from Center for Disease Control (Hubei, China). All mice were housed in colony cages in a pathogen-free environment with the temperature maintained at 21–23°C and relative humidity at 50–60% and were under a 12 h:12 h light: dark cycle. For fasted and refed treatment, the fasted group mice were fasted at 19:00 pm and refed with chow diet at 7:00 am and the refed group was fasted at 7:00 am and refed with Rodent diet with 10% kcal fat (Research Diets, D12450B) at 19:00 pm for 3 days. For diet-induced obesity experiments, all mice were fed a high-fat diet (HFD) (Research Diets, D12492) for 15 weeks and divided into two groups. Mice were injected 0.5 mg/kg si-Control or si-*Usp20* every week for 7 weeks. Metabolic rates were measured at the fourth week by a comprehensive lab animal monitoring system (Columbus Instruments). Measurements of VO_2_ was recorded over 2 days. The analyses of oxygen consumption and activities in mice were performed by CalR-analysis of covariance (32). Body composition of mice was measured by time-domain nuclear magnetic resonance with Bruker Minispec LF50 (Bruker Optics). The rectal temperatures of the mice were determined by a rectal probe attached to a digital thermometer (Physitemp). For cold exposure test, mice were housed at 4°C for 4 h, and the mice rectal temperature was measured every hour. For thermoneutral experiment, mice were housed at 22°C or 30°C for 3 days. For succinate stimulates thermogenesis experiment, WT mice fed with or without 2% sodium succinate in water for 8 weeks (n = 6 per group). *Ldlr*^−/−^ mice were purchased from GemPharmatech, and mice were fed 1.25% cholesterol and 0.5% sodium cholate diet (Research Diets, D12109C) for 8 weeks. For the siRNA and statin cotreatment experiment, mice were injected with si-Control or si-*Usp20* once and then orally gavaged with 0.5% methylcellulose solution with or without 15 mg/kg/day atorvastatin for 3 days. All mice were starved for 4 h before euthanasia and analysis. All animal care and use procedures approved by guidelines of the Institutional Animal Care and Use Committee of Wuhan University.

### Reagents

We obtained protease inhibitor cocktail (P8340), paraformaldehyde (P6148), and Sudan IV (198102) from Sigma-Aldrich; phenylmethylsulfonyl fluoride (HY-B0496) and MG132 (HY-13259) were from MCE. ALLN (208719) and pepstatin A (516,481 mol/L) were from Calbiochem. Dithiothreitol (T5370) was from Targetmol. PBS (120539) was from Monad. Succinate (485349) was from Sigma.

### Cell culture

Hepa1-6 was cultured in DMEM containing 100 units ml^−1^ penicillin and 100 μg/ml streptomycin sulfate supplemented with 10% fetal bovine serum. Primary hepatocyte was isolated from 8-week-old male C57BL/6J mice fed chow diet. To disperse hepatocytes, mouse liver was perfused with collagenase (Sigma, C0130). Primary hepatocytes were collected by centrifugation and was cultured in Medium199 (Life Technologies) containing 100 units ml^−1^ penicillin and 100 μg/ml streptomycin sulfate supplemented with 10% fetal bovine serum in collagen-coated petri dishes. All the cells were grown in a monolayer at 37°C in 5% CO_2_.

### Immunoblotting

Cells or tissues were collected and homogenized with RIPA buffer (50 mM Tris-HCl, pH 8.0, 150 mM NaCl, 2 mM MgCl_2_, 1.5% NP-40, 0.1% SDS, and 0.5% sodium deoxycholate) supplemented with protease inhibitor cocktail (10 μg ml^−1^ leupeptin, 1 mM phenylmethylsulfonyl fluoride, 5 μg/ml pepstatin, 25 μg/ml N-acetylleucinal-leucinal-norleucinal, and 1 mM dithiothreitol). Protein concentrations were determined using BCA kit (23225 Thermo Fisher Scientific). Samples were mixed with the membrane solubilization buffer (62.5 mM Tris-HCl, pH 6.8, 15% SDS, 8 M urea, 10% glycerol, and 100 mM DTT) plus the 4 × loading buffer (150 mM Tris-HCl, pH 6.8, 12% SDS, 30% glycerol, 6% 2-mercaptoethanol, and 0.02% bromophenol blue) and incubated for 30 min at 37°C. The equal amounts of total proteins were resolved by SDS–PAGE and transferred to PVDF membranes. Immunoblots were blocked with 5% nonfat milk in tris-buffered saline containing 0.075% Tween 20 (TBST), probed with indicated primary antibodies overnight at 4°C. After washing in TBST three times, blots were incubated with horse radish peroxidase-conjugated secondary antibodies (1:5,000) diluted in TBST supplemented with 5% skim milk for 1 h at room temperature and followed by at least three washes with TBST. Immunoreactivity was developed with enzymatic detection using Pierce ECL Plus Western blotting substrate (Thermo Fisher Scientific).

Primary antibodies used for immunoblots were as follows: mice monoclonal antibody (clone AC-15) against β-actin (Sigma, A1978, diluted 1:10,000) were prepared from hybridomas (ATCC). Rabbit polyclonal antibodies against HMGCR (diluted 1:1,000) were prepared in our laboratory. Rabbit polyclonal anti-USP20 antibody (diluted 1:1,000) was purchased from Bethyl (A301-189A). Anti- UCP1 (23673, diluted 1:1,000) and FASN (66591, diluted 1:1,000) antibodies were obtained from Proteintech.

### Glucose tolerance and insulin tolerance tests

Ahead of studies, mice were injected with si-Control or si-*Usp20* RNA for 6 weeks for glucose tolerance tests (GTT) or 7 weeks for insulin tolerance tests (ITT). In GTT studies, glucose (2 g/kg) was injected intraperitoneally into mice. For ITT, mice received an intraperitoneal injection of insulin (0.75 U/kg). Tail-blood glucose levels were measured at 0, 15, 30, 60, 90, and 120 min after challenge using the Onetouch Ultra blood glucose monitoring system (Johnson).

### Biochemical assays

Blood glucose was measured from the tail vein using the Onetouch UltraEasy blood glucose monitoring system (Johnson). Mice blood was collected by retro-orbital bleeding. The livers were homogenized, and supernatants were collected for lipid extraction. Total cholesterol (TC) and triglyceride (TG) levels were determined by the TC kit and TG kit according to the manufacturer’s instructions (Kehua Bio-engineering). The serum levels of ALT and AST were determined according to the manufacturer’s instructions (C009-2-1 for ALT, C010-2-1 for AST, NJJCBIO). The serum level of insulin was measured by the mice insulin immunoassay kit (MS100, EZassay). Serum TNFα and creatinine were measured using ELISA kits (SCA133Mu for TNFα, CEV806Ge for creatinine, Cloud Clone). Free fatty acid was enzymatically measured with a kit (NEFA, Wako, Japan). To measure the biliary concentration, 5 μl of bile was mixed with 45 μl Milli Q water and then extracted with 200 μl chloroform/methanol (2:1). The organic phase and aqueous phase were separated and dried and dissolved in ethanol and Milli Q water, respectively. The organic phase was determined by TC and TG kit (Shanghai Kehua bio-engineering Co.) Biliary phospholipid was measured using commercial assay kits (WAKO LabAssay, No. 296–63801).

### Quantitative PCR

Total RNA was extracted from homogenized liver or cells using Trizol Reagent (Invitrogen) following the manufacturer’s instructions. Total RNA was digested by RNase-free DNase I (Promega). Synthesis of cDNA was performed using 2 μg total RNA from each sample using M-MLV Reverse Transcriptase (Promega). qPCR was carried out using the Hieff qPCR SYBR Green Master Mix (Cat No:11201ES08; Yeasen, Shanghai, China) and analyzed on a Bio-Rad CFX96 apparatus (Bio-Rad).

### Atherosclerotic plaque analysis

Eight-week-old male *Ldlr*^−/−^ mice were given weekly injections of 0.5 mg/kg of siRNA, and all mice were fed a 1.25% cholesterol and 0.5% sodium cholate diet (Research Diets, D12109C) for 8 weeks. After 4 h of fasting, the mice were euthanized by cervical dislocation and perfused with approximately 20 ml of PBS via left ventricular puncture. Liver, brown adipose tissue (BAT), and epididymal white adipose tissue (eWAT) were removed intactly and fixed in 4% paraformaldehyde at 4°C for 24 h. The aorta was stripped along the spine under an Olympus SZX16 stereomicroscope. Then the perivascular adipose tissue was removed to expose the aortic arch and the cephalic brachial artery, the left carotid artery, and the left subclavian artery. Blue background was padded under the aortic arch before photographs were taken.

For the aortic arch stanning, the heart was removed intactly, the periaortic fatty tissue was removed, the aorta was cut below the bifurcation of the aorta to the iliac arteries, the renal arteries were cut close to the kidneys, and the three branching blood vessels of the neck were cut from the distal end. The intact heart and the entire aorta were fixed in 4% paraformaldehyde at 4°C for 24 h. After removal of adjacent adipose and connective tissue, the aorta was cut longitudinally (from the root of the aorta to the bifurcation of the iliac arteries, along with the three branching vessels) and fixed with fine needles. Then the entire aorta was stained with a modified Oil red O kit (Biyuntian). Photographs were taken for imaging under an Olympus SZX16 stereomicroscope. Aortic plaque distribution was quantified by Image J.

### Tissue sections and staining

Mouse heart, liver, eWAT, and BAT were fixed in 4% paraformaldehyde at 4°C for 24 h and sectioned by paraffin embedding. Heart was stained with hematoxylin and eosin staining (HE) and Oil Red O. Liver was stained with HE, Oil Red O, and Masson. BAT and eWAT were stained with HE. Photographs were taken using a Leica fully automated section scanning microscope with 20 × magnification.

### Succinate analysis

The LC-MS/MS system was used to analyze the succinate level of serum and liver. Briefly, to 50 μl of serum, add 150 μl of ddH_2_O containing 5 mg of succinic acid-1, 4–13C (sigma, 485349) as the internal standard, for liver tissue, and 100 mg of one sample was homogenized in 200 μl ddH_2_O with 5 mg succinic acid-1, 4–13C. 800 μl of 1:1 solution of methanol and acetonitrile was used to extract succinate. Samples were frozen in liquid nitrogen and sonicated three times for 5 min each time. After centrifugation at 13,000 rpm at 4°C, 850 μl of the supernatant was taken to dry and dissolved with 100 μl of 50% acetonitrile by sonication as the loading sample. The samples were diluted 10 times with 50% acetonitrile and analyzed by LC-MS/MS (UPLC: Waters, ACQUITY UPLC H-Class; MS: AB SCIEX, QTRAP5500 hybrid dual-quadrupole ion-trap mass Spectrometer). The analytical condition was as follows: UPLC: column, Waters Acquity UPLC BEH Amide Column (2.1 × 100 mm, 1.7 μm particles) at 40°C. Solvent A was 20 mM ammonium acetate, 20 mM ammonia water, 5% ACN, 95% H_2_O, pH = 9.0, and solvent B was 100% acetonitrile. The flow rate was 0.5 ml/min with the following gradient: 0–1 min 95% B, 1–2.5 min from 95% B to 40% B, 2.5–4.5 min 40% B, 4.5–5.0 min from 40% B to 95% B, and 5–6 min 95% B. The total gradient is 6 min. The mass spectrometer was operated in negative ion mode with a source voltage of −4,500 V, curtain gas setting was 35, and collision gas setting was medium. Multiple reaction monitoring was used to quantify metabolites. Peak determination and integration were performed by Analyst 1.7.0 and OS 1.4.0 SCIEX software.

### Statistical analysis

GraphPad Prism 9 software was used for all statistical analyses. The data were presented as means ± SEM and analyzed using an unpaired two-tailed Student's *t* test or a one-way ANOVA with Dunnett's multiple comparisons test, if appropriate. Statistical tests were justified as necessary for each figure. The statistical significance level was chosen at *P* < 0.05. The sample sizes, statistical tests, and *P* values for each experiment are shown in the image legends. Considering the effect of different time periods on the movement of mice, we compared the movement of mice in the si-*Usp20* group and control group by analysis of covariance with the period as a covariate. Experiments on mice were carried out once with the specified n and biological replicates.

## Results

### Identification of effective siRNA molecules targeting *Usp20*

**Fig. 1:**
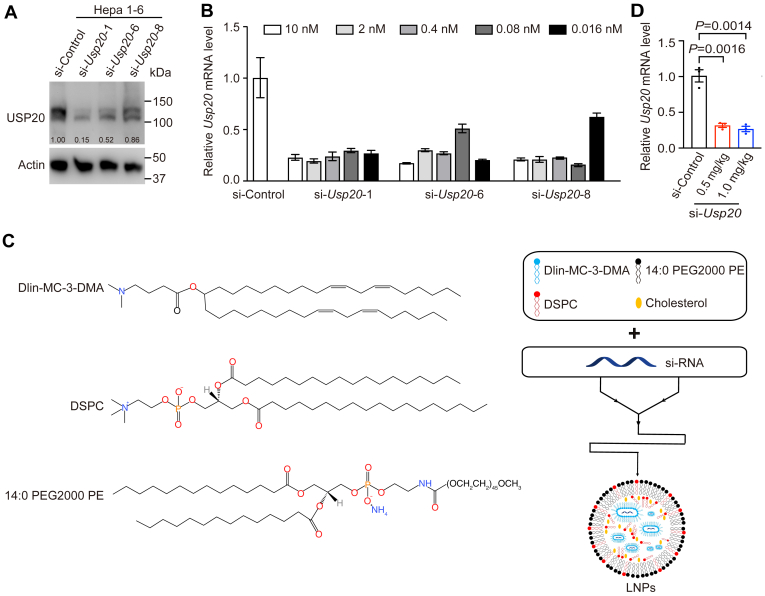
Identification of effective siRNA molecules targeting *Usp20*. A, B: The mRNA and protein levels analyzed 24 h posttransfection of indicated siRNAs at a concentration of 10 nM in Hepa1-6 cells. C: Chemical structure of major lipids used for LNPs and schematic of LNP-siRNA formulation process. D: The relative *Usp20* mRNA levels analyzed 24 h post intravenous administration of LNPs at a dose of 0.5 mg/kg and 1.0 mg/kg si-*Usp20* in C57BL/6 mice.

A series of siRNAs targeting *Usp20* were designed using bioinformatic analysis. To reduce immunogenicity and enhance stability, some nucleotides in the siRNA were modified with 2′-O-methoxyethyl and thiol groups, and the siRNA had a two deoxythymidines overhanging on the 3′ end (supplemental Fig. S1A) (33). The effectiveness of each siRNA at a concentration of 10 nM was evaluated (supplemental Fig. S1B). Three siRNAs were found to cause significant knockdown of *Usp20* in Hepa1-6 cells (Fig. 1A and supplemental Fig. S1C). A dose-response analysis was then carried out, and the siRNA that exhibited the greatest potency in targeting *Usp20* (si-*Usp20*) was selected for in vivo study. A very low dose (16 pM) of si-*Usp20*-1 resulted in more than 70% reduction in *Usp20* mRNA (Fig. 1B). USP20 protein levels were largely depleted 24 h after transfection (Fig. 1A). Si-*Usp20*-1 was able to effectively decrease *Usp20* mRNA and protein levels in primary hepatocytes (supplemental Fig. S1C, D).

To investigate the silencing activity of si-*Usp20* in vivo, siRNAs were formulated in ionizable cationic LNPs using the NanoAssemblr™ (Fig. 1C) (34). The lipid compositions were based on the D-lin-MC-3-DMA which is approved by the FDA. Next, the particle size of si-*Usp20* LNPs was determined using dynamic light scattering, which revealed an average size of approximately 77.89 ± 0.90 nm, with a polydispersity index of 0.07 ± 0.01 (supplemental Fig. S1E, F). The encapsulation efficiency reached 88.14%, suggestive of successful formulation (supplemental Fig. S1E). To verify the knockdown efficiency in vivo, si-*Usp20* LNPs were administered at 0.5 or 1.0 mg/kg via tail vein injections. At 24 h postadministration, livers were harvested to measure *Usp20* mRNA levels. As shown in Figure 1D, about two thirds of *Usp20* mRNA was silenced after 0.5 mg/kg siRNA-LNP treatment.

### Silencing of *Usp20* alleviates obesity caused by long-term HFD

**Fig. 2:**
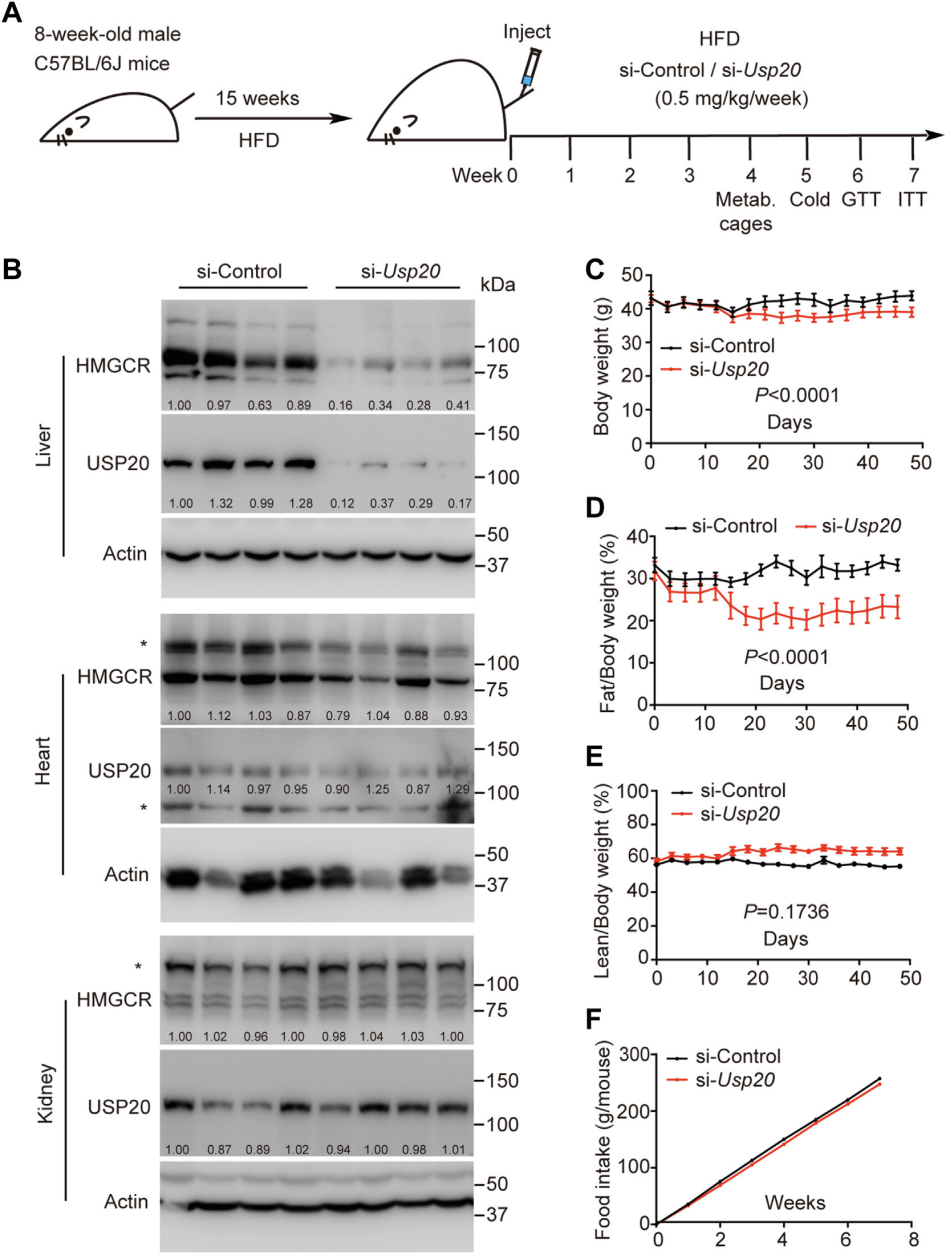
Silencing of *Usp20* alleviates obesity caused by long-term high-fat diet. A: Schematic of treatment with *Usp20* siRNA. Eight-week-old male C57BL/6J mice were randomly grouped, fed the HFD diet for 15 weeks and then injected with 0.5 mg kg^−1^ si-Control or si-*Usp20* RNA weekly for another 7 weeks. B: Immunoblotting analysis of HMGCR and USP20 in the indicated tissue, asterisk indicates nonspecific band. C-E: Body weight and whole-body composition of the mice after 7 weeks of HFD feeding. F: Food intake of the mice during the whole experiment. Data were expressed as means ± SEM. Data were analyzed by unpaired two-tailed Student’s *t* test.

To test whether knocking down *Usp20* can treat metabolic diseases, we fed mice a HFD. When the body weight reached ∼40 g, mice were randomly divided into two groups and injected with si-Control or si-*Usp20* (0.5 mg/kg/week), respectively (Fig. 2A). After 8 weeks, the mice were euthanized. si-*Usp20* effectively reduced USP20 protein levels to ∼25% in the liver but has no effect in the kidney or heart. The level of HMGCR in the liver was also significantly reduced to ∼30% in the si-*Usp20* group compared to the si-Control group (Fig. 2B), demonstrating that the LNP-encapsulated siRNAs specifically targeted the liver. Compared with the control group, si-*Usp20* decreased body weight and reduced fat composition by ∼10% without altering food intake (Fig. 2C–F).

### si-*Usp20* mice exhibited higher energy expenditure

**Fig. 3:**
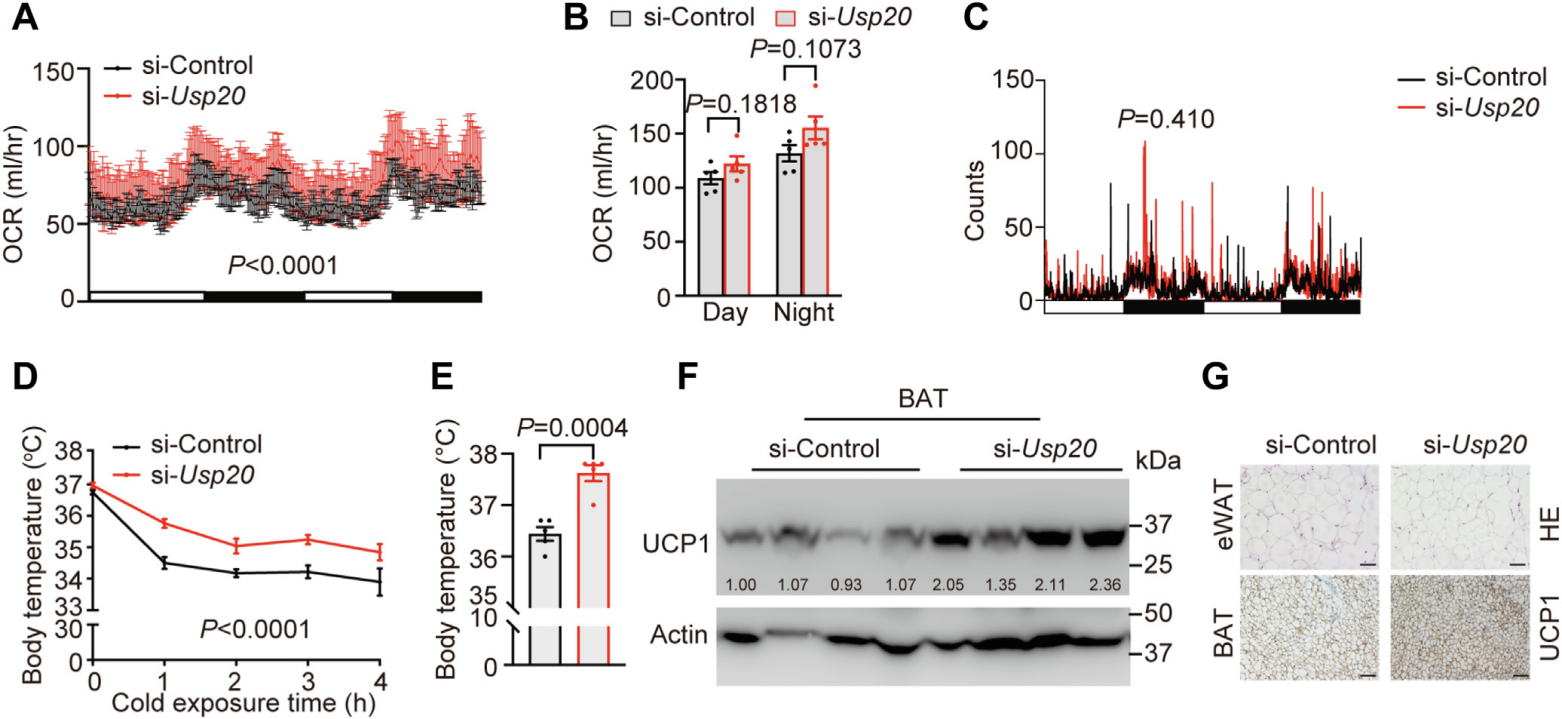
si-*Usp20* mice exhibited higher energy expenditure. A: Whole-body oxygen consumption rate (OCR) of mice (n = 5 per group) during day and night. B: The average oxygen consumption of mice (n = 5 per group) in a day (white box represents am 07:00-pm 19:00) or a night (black box represents pm 19:00-am 07:00). C: Movement as determined by metabolic cages (n = 5 per group). D: Rectal temperature of mice (n = 5 per group) at different times after 4°C cold exposure. E: Rectal temperature of the mice (n = 5 per group). F: Immunoblotting analysis of BAT samples (n = 4 per group). G: Representative HE is staining of eWAT and UCP1 staining of BAT sections. Scale bars, 50 μm. Data are expressed as mean ± SEM, data were analyzed by unpaired two-tailed Student’s *t* test (B, E), two-way ANOVA (A, D) and ANCOVA (C). ANCOVA, analysis of covariance

To examine the effect of siRNA on fat metabolism, we performed metabolic analysis on HFD-fed mice. Metabolic cage analysis showed a tendency for higher oxygen consumption in mice injected with si-*Usp20*, but there was no difference in the movement frequency, which indicated that the increase in metabolic levels was not due to increased physical activity (Fig. 3A–C). When exposed to a 4°C environment, mice with silenced *Usp20* maintained a higher body temperature (Fig. 3D, E). BAT is an important organ for nonshivering heat thermogenesis. UCP1 is a mitochondrial inner membrane transmembrane protein that increases the heat production and accelerate fat consumption (35). UCP1 was significantly upregulated in BAT in the si-*Usp20* group (Fig. 3F, G).

In order to clarify the relationship between *Usp20* and *Ucp1* in BAT, we detected the expression of *Usp20* under different dietary conditions or different temperature environments. *Usp20* expression level is very low in adipose tissue (12), and the mRNA level of *Usp20* is not affected by fasted-refed treatment or environmental temperature (supplemental Fig. S2A, B). The si-*Usp20* reduced the expression level of *Usp20* in the liver by ∼ 70% but did not alter *Usp20* expression in adipose tissues (supplemental Fig. S2C). We detected the expression of thermogenesis-related genes in BAT and eWAT. The expression of *Ucp1*, *Pgc1a*, and *Cox8b* was increased in BAT (supplemental Fig. S2D), and other genes including *Prdm16*, *Cidea*, *beta-adrenergic receptor*, and *Atp2a2* were unchanged when *Usp20* was silenced (supplemental Fig. S2E, F).

### Silence of *Usp20* improves metabolic profile

**Fig. 4:**
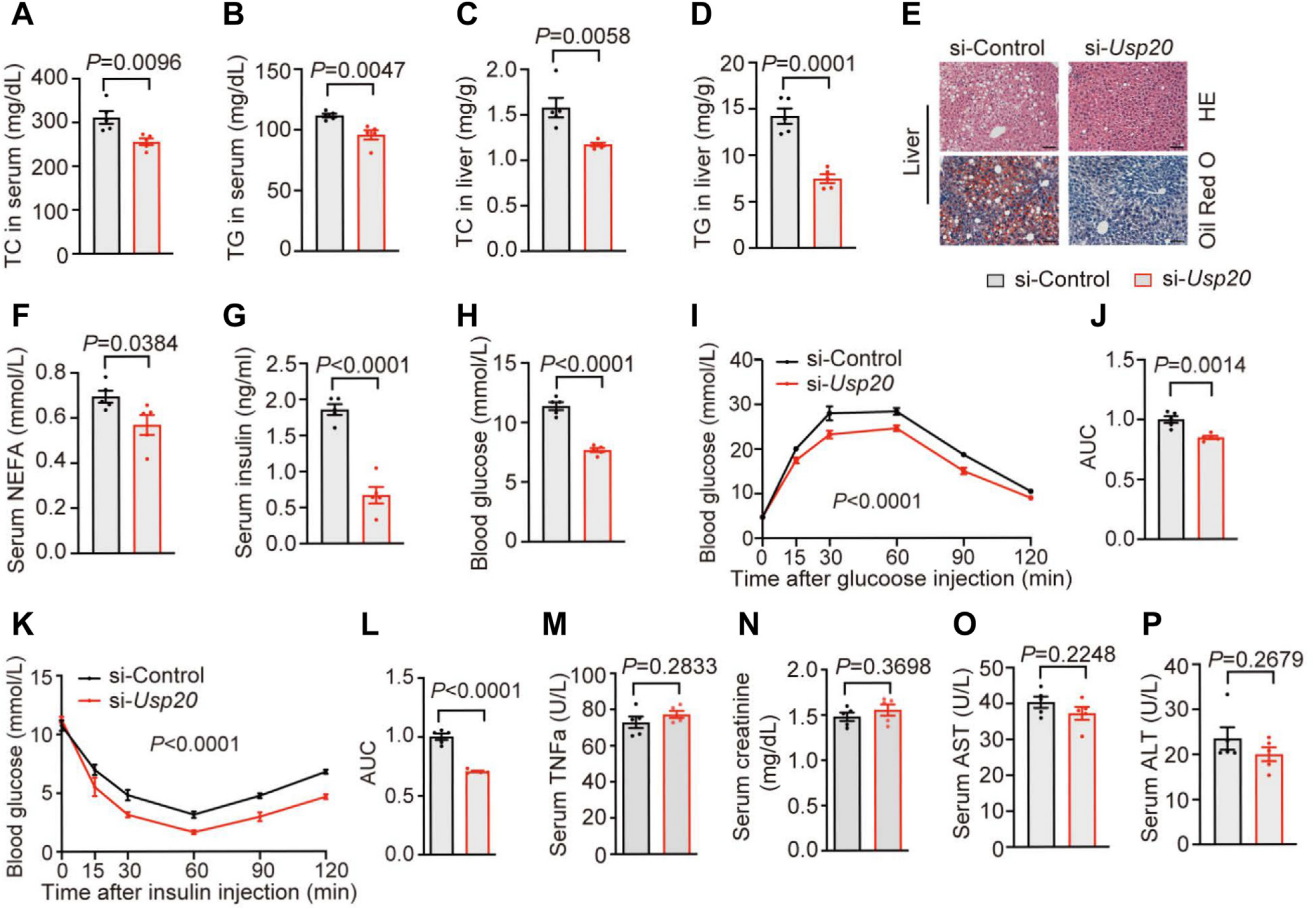
Silence of *Usp20* improves metabolic profile. A-D: TC and TG levels in serum and liver. E: Representative HE and Oil Red O staining of liver sections. Scale bars, 50 μm. F,G: NEFA and insulin levels in serum. H: Blood glucose level. I-L: Glucose tolerance and insulin tolerance after six weeks of HFD feeding (n = 5 per group). M-P: Serum levels of TNFα (M), creatinine (N), AST (O) and ALT (P). Data are expressed as mean ± SEM, data were analyzed by unpaired two-tailed Student’s *t* test (A–D, F–H, J, L–P) or two-way ANOVA (I, K).

To further explore the metabolic parameters in si-*Usp20*-injected mice, we tested lipid levels in liver and serum of mice fed HFD. The si-*Usp20* group displayed lower lipid levels than the control group (Fig. 4A–E). Meanwhile, circulating NEFA, insulin, and glucose levels were significantly lower in si-*Usp20* mice than controls (Fig. 4F–H). Furthermore, the GTT and ITT showed that glucose clearance and insulin sensitivity were improved in si-*Usp20* mice (Fig. 4I–L). No significant changes were found in the levels of TNFα, creatinine, AST, and ALT, suggesting siRNA treatment did not result in inflammation, renal toxicity or liver damage (Fig. 4M–P).

### Ablation of hepatic *Usp20* increases energy expenditure and UCP1 expression

**Fig. 5:**
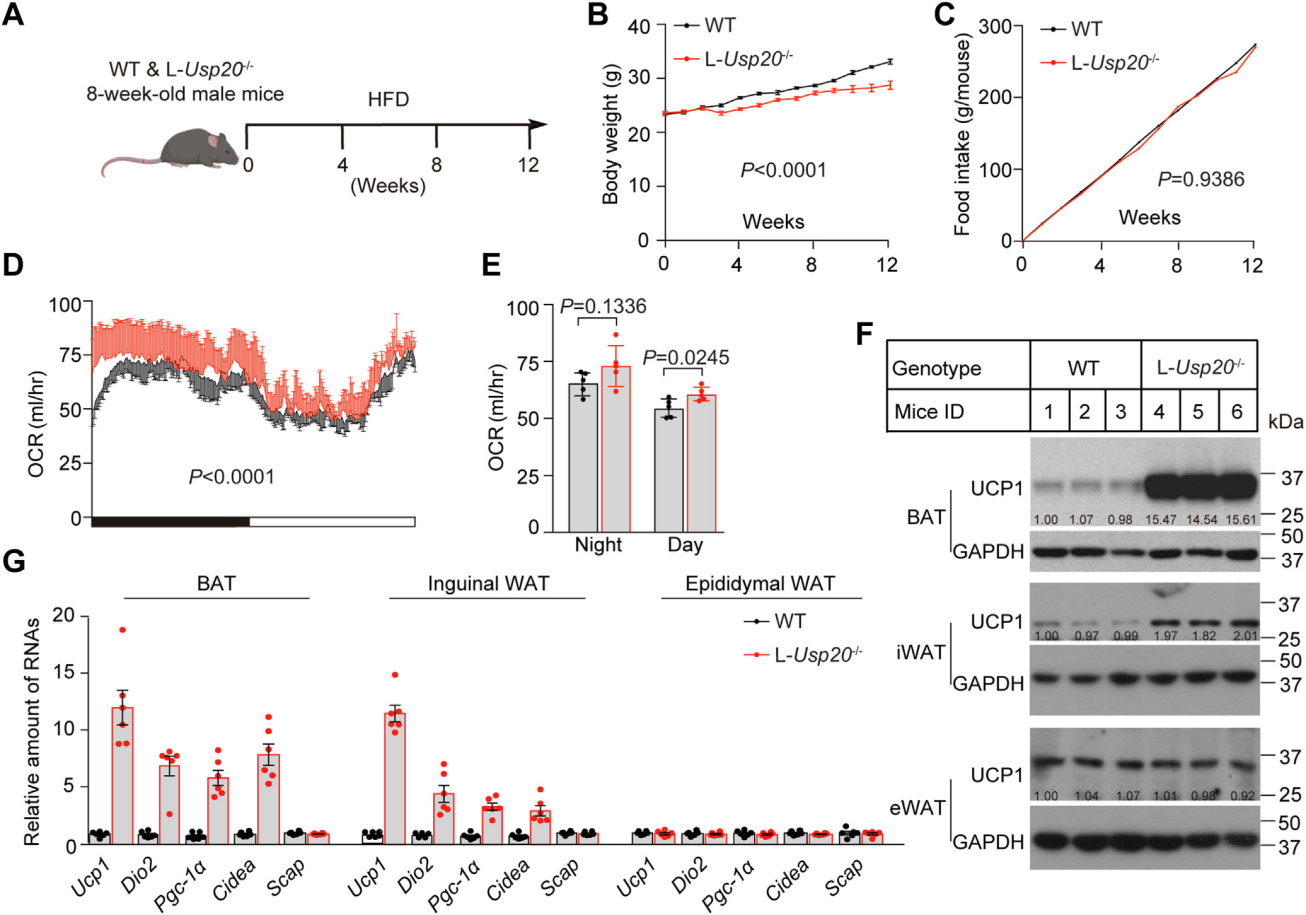
Ablation of hepatic *Usp20* increases energy expenditure and UCP1 expression. A: Schematic of WT and L-*Usp20*^−/−^mice fed with high-fat diet for 12 weeks (n = 6 per group). B: Body weight of the mice over 12 weeks. C: Cumulative food intake of the mice over 12 weeks. D: Whole-body oxygen consumption of mice in 1 day (white box represents am 07:00-pm 19:00) or a night (black box represents pm 19:00-am 07:00). E: Average oxygen consumption over the day and night. F: Immunoblot of UCP1 protein in different adipose tissues, and each group contains randomly three biological replicates. G: Relative amounts of mRNA of thermogenesis-related genes in adipose tissue. Data are expressed as mean ± SEM, and *P* values were calculated by unpaired two-tailed Student’s *t* test.

To investigate the effect of the liver *Usp20* gene on energy metabolism, we fed L-*Usp20*^−/−^ mice a HFD and monitored metabolic levels (Fig. 5A). The liver-specific deletion of *Usp20* slowed weight gain without altering food intake (Fig. 5B, C). L-*Usp20*^−/−^ mice exhibited higher energy expenditure than WT mice (Fig. 5D, E). Notably, the expression of UCP1 in BAT and inguinal WAT (iWAT), but not eWAT, was dramatically increased in the L-*Usp20*^−/−^ mice (Fig. 5F, G).

**Fig. 6:**
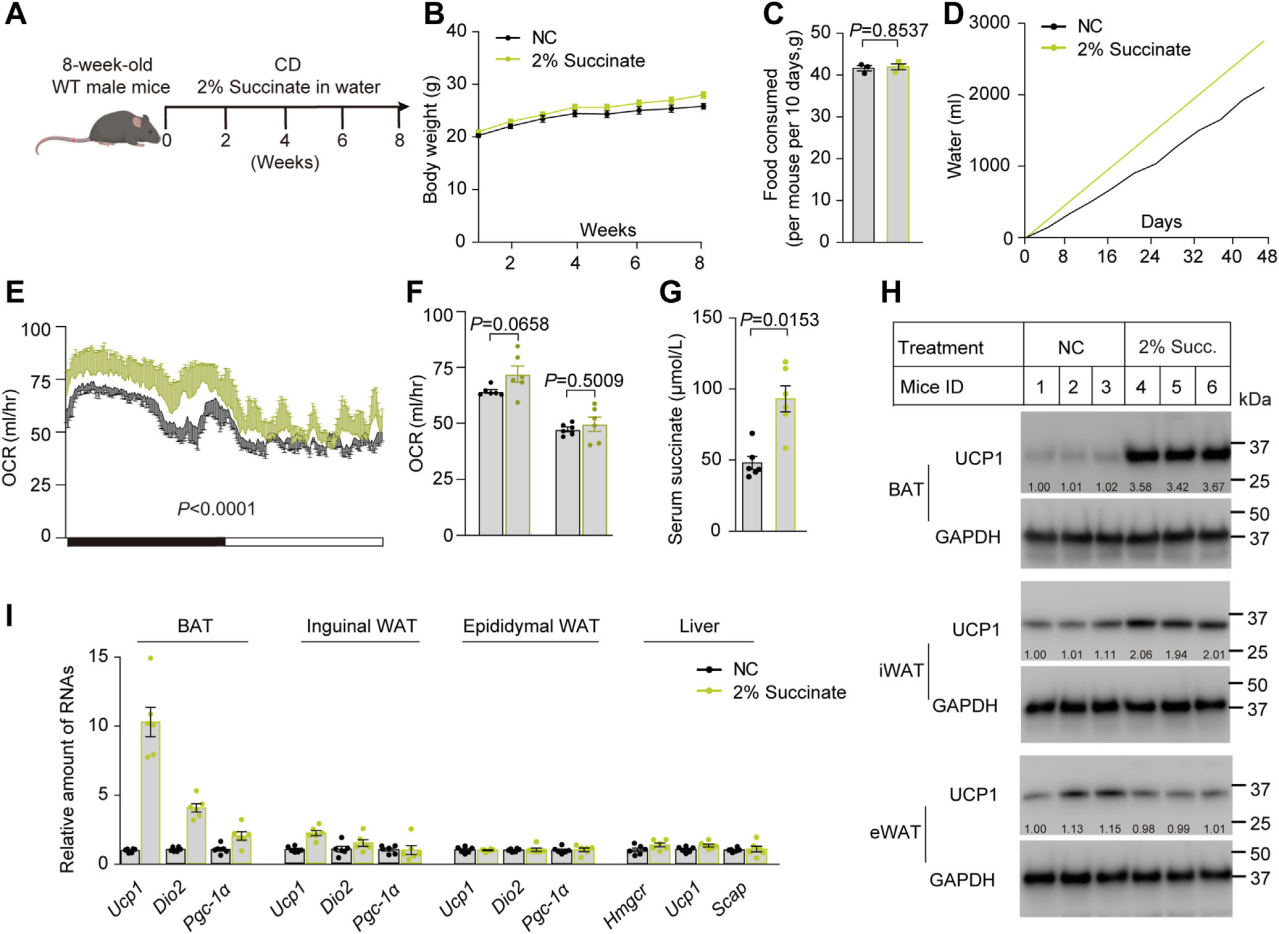
Succinate increases BAT thermogenic efficiency. A: Schematic of WT mice fed with or without 2% sodium succinate in water for 8 weeks (n = 6 per group). B: Body weight of the mice over 8 weeks. C: The total food intake of each mouse in 10 consecutive days. D: Cumulative water intake over 8 weeks. E: Whole-body oxygen consumption of mice in 1 day. F: Average oxygen consumption over the day and night. G: Succinate level in serum of the mice. H: Immunoblot of UCP1 protein in different adipose tissues, each group contains randomly three biological replicates. I: Relative amounts of mRNA of thermogenesis related genes in adipose tissue and lipid synthesis-related genes in liver. Data are expressed as mean ± SEM, and data were analyzed by unpaired two-tailed Student’s *t* test.

It is known that *Usp20* deficiency increases succinate that can stimulate UCP1-dependent thermogenesis (12, 36, 37, 38). To confirm that succinate increase UCP1 level that have been shown before, we added 2% sodium succinate to the drinking water of mice and examined the metabolic profile (Fig. 6A). Succinate did not increase the body weight and food intake but increased the water intake of mice (Fig. 6B–D). Similar to L-*Usp20*^−/−^ mice, the mice that received sodium succinate had higher oxygen consumption and higher circulating succinate concentrations (Fig. 6E–G). The gene expression and protein level of UCP1 in BAT and iWAT, but not eWAT, were significantly increased (Fig. 6H, I). These data suggest that silencing of hepatic *Usp20* increases heat production through succinate-induced elevation of UCP1 in BAT and iWAT.

### Silencing *Usp20* alleviate atherosclerosis

**Fig. 7:**
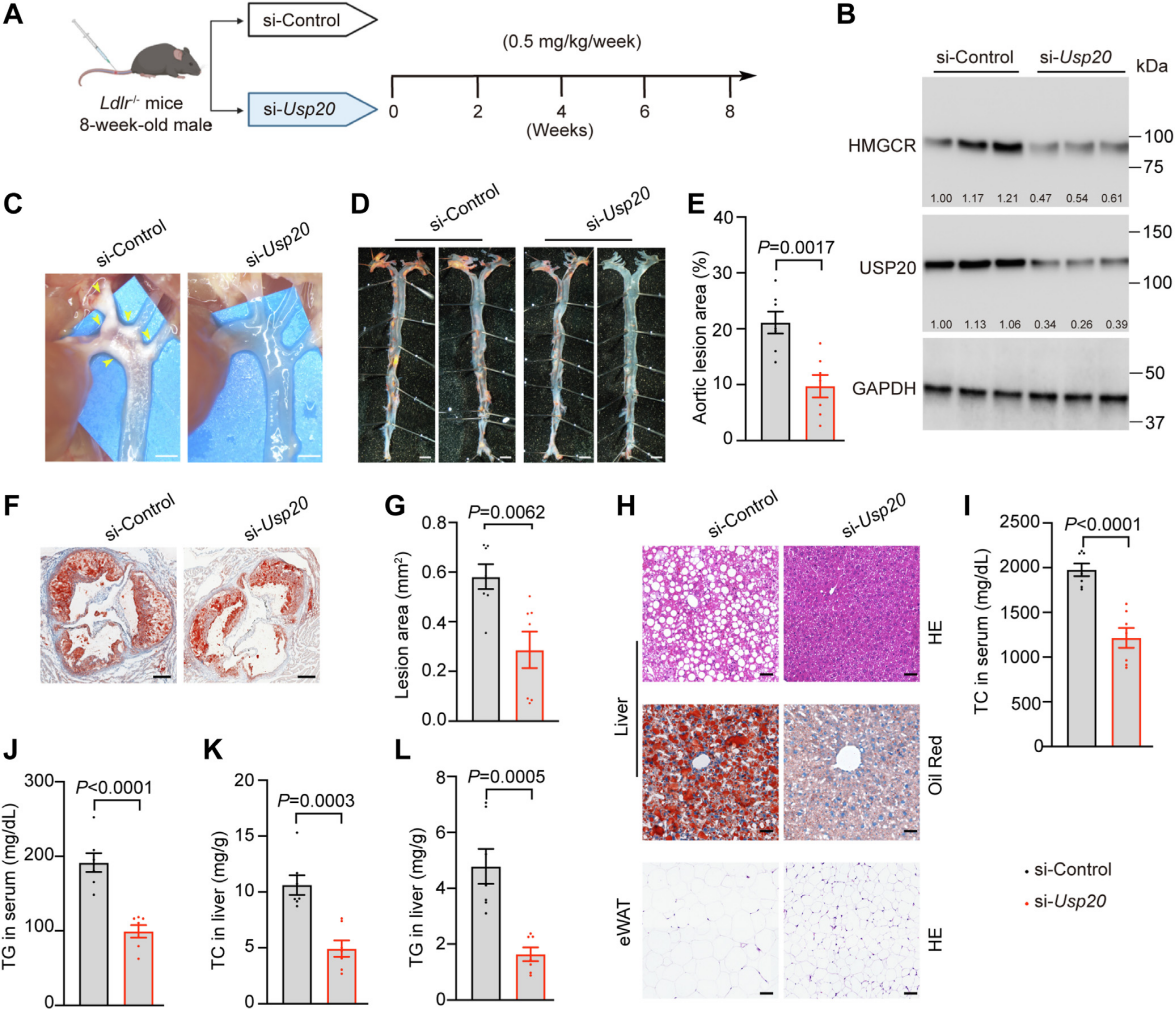
Silencing *Usp20* alleviate atherosclerosis. A: Schematic of treatment with *Usp20* siRNA. Eight-week-old male *Ldlr*^−/−^ mice were injected with 0.5 mg kg^−1^ si-Control or different si-*Usp20* RNA weekly for 8 weeks (n = 7 per group). B: Immunoblotting analysis of liver samples contains randomly three biological replicates. C: Aortic arch in situ images. Scale bars, 1 mm. D: En face lipid staining of aortas scale bars, 1 mm. Quantifications of atherosclerotic lesions shown in (E). F: Oil red O staining of aortic arch root sections, scale bars, 100 μm and quantification of lesion area in (G). H: Liver and eWAT sections stained with HE or Oil red O, Scale bars, 50 μm. I-L: TC and TG levels in serum and liver. Data are presented as mean ± SEM. Data were analyzed by unpaired two-tailed Student’s *t* test.

To examine the effect of *Usp20* knocking down on hyperlipidemia and atherosclerosis, we injected the LNP-encapsulated siRNA into *Ldlr*^−/−^ mice and fed them high-fat and high-cholesterol diet for 8 weeks (Fig. 7A). The mice injected with control siRNA and *Usp20* siRNA (efficiently knock down *Usp20*) showed similar food intake and bodyweight gain, and tissue weight of organs such as liver and kidney also showed no difference (supplemental Fig. S3A–D). Next, we assessed the effect of siRNA on different tissues. The group treated with si-*Usp20* had lower USP20 level and HMGCR level in the liver, as USP20 was reduced to ∼30%, and HMGCR was decreased to ∼50% in the *si-Usp20* group (Fig. 7B). The atherosclerotic plaque accounted for ∼20% of the total aortic area in the control siRNA group (supplemental Fig. S3E). In contrast, the si-*Usp20* dramatically reduced plaque area to ∼10% (Fig. 7C–E). We performed in situ analysis and root staining analysis of the aortic arch in the atherosclerosis experiments (Fig. 7F, G). Compared with the control group, si-*Usp20* decreased serum TC and TG levels by∼50% (Fig. 7I, J). Liver section staining and biochemical analysis revealed that the lipid level in the liver was decreased by si-*Usp20* (Fig. 7H, K, L). Therefore, these results demonstrated that silencing *Usp20* can prevent atherosclerosis.

The mice injected with siRNA showed similar AST and ALT levels, indicating that there were no obvious symptoms of inflammation in the liver (supplemental Fig. S3F, G). Comparing the si-*Usp20* group with si-Control group, there are no difference in kidney damage and inflammation factors (supplemental Fig. S3H–J). To examine the effect of siRNA on adipose tissue, we tested the *Usp20* and *Ucp1* expression level. Silencing of *Usp20* led to the increase of UCP1 and energy expenditure (supplemental Fig. S3K, L, O), proving that knocking down *Usp20* in liver can increase adipose thermogenesis. We have measured succinate levels in serum and liver. Knockdown of *Usp20* indeed increased succinate levels in serum and liver in *Ldlr*^−/−^ mice, consistent with UCP1 expression in adipose tissue (supplemental Fig. S3M, N). Besides, we examined gene expression in BAT, eWAT, and liver; silencing of *Usp20* led to the increase expression of *Ucp1* mRNA level in BAT, lowered the mRNA levels of *Scd-1*, *Fasn*, and *Acs* in liver and expression of lipid metabolism and other thermogenesis, and inflammatory factors related genes were all similar among the two groups (Supplemental Fig. S3O–Q). In conclusion, inhibition of *Usp20* by an LNP–siRNA complex can alleviate hyperlipidemia and prevent atherosclerosis.

### Silence of *Usp20* inhibit statin-induced upregulation of HMGCR expression

Statins are commonly used cholesterol-lowering drugs, but they can cause activation of the SREBP pathway and subsequently upregulate HMGCR expression. In order to verify whether knockdown of *Usp20* has an effect on statin-induced HMGCR expression, we conducted a mice experiment with statin and *Usp20* siRNA. Mice were injected with si-*Usp20* and then orally gavaged with 15 mg/kg/day atorvastatin for 3 consecutive days. Atorvastatin dramatically increased HMGCR level by > 20-fold that may cause statin-resistance and toxic effect. The si-*Usp20* completely blunted statin-induced HMGCR increase (supplemental Fig. S4A). Coadministration of atorvastatin and si-*Usp20* showed the trend of less lipid levels (supplemental Fig. S4B, C).

## Discussion

LNPs are lipid vesicles that have a homogeneous lipid core. These vesicles are commonly employed in the transfer of small molecules and nucleic acids (39). The two main areas where siRNA drugs are now being explored are oncology and rare diseases, and numerous siRNA treatments for uncommon disorders have already obtained commercial approval (40). Hypercholesterolemia is treated with siRNA targeting proprotein convertase subtilisin/kexin 9 (41). Because of their high specificity and efficiency, siRNA therapeutics are gaining popularity, and therapeutic strategies based on siRNA are being developed to treat a variety of disorders (42). However, just 1–4% of the RNA can escape from the endosomes, demonstrating a limited ability to escape from the endosomes. Because the bulk of current LNPs target the liver, the challenge of targeted and specific delivery in organs other than the liver must be addressed promptly (43). Importantly, the higher complexity of LNP synthesis makes it difficult to manufacture and increases its toxicity, which may limit its use in clinical applications (44).

Statins, which inhibit HMGCR enzymatic activity, are the most commonly used cholesterol-lowering drugs (45). However, doubling the statin dose only reduces LDL-cholesterol by 6%, and statins were intolerant in 9.1% of patients (46). Statins can cause liver damage (47) and muscular problems (48) among other side effects. Several investigations have shown that statins increase the protein level of HMGCR by blocking ubiquitination, resulting in the development of statin resistance or a rapid increase in cholesterol levels after drug discontinuation (49). Atorvastatin dramatically increased HMGCR level by >20-fold that may cause statin-resistance and toxic effect (50, 51). In our study, the si-*Usp20* completely blunted statin-induced HMGCR increase and showed the trend of less lipid levels (supplemental Fig. S4A–C).

Our previous study found that the mammalian target of rapamycin 1-USP20-HMGCR pathway regulates cholesterol synthesis de novo in the feeding state and accelerates the conversion from carbohydrate to lipid. Endogenous cholesterol production can be greatly reduced by inhibiting USP20 function or reducing its expression in the liver. We used LNPs to coat targeted siRNA to specifically reduce the expression of liver *Usp20* in mice to test the effect of the RNAi approach on *Usp20*.

We examined the effects of si-*Usp20* injection on different tissues of mice. After injection of si-*Usp20*, the level of HMGCR protein was decreased to ∼50% (Figs. 2B and 7B), and the fatty acid synthesis pathway was downregulated in obesity resulting from a HFD (supplemental Fig. S3Q). Meanwhile, no suppression of *Usp20* expression in other tissues was detected, proving that LNPs have high liver-targeting specificity, and the metabolic beneficial effects of *Usp20* siRNA were on-target (Fig. 2B, supplemental Figs. S2C and S3C, D). The mice injected with si-*Usp20* lost weight without reducing their food consumption significantly; therefore, the indications of insulin resistance were treated, and their metabolic level was increased (Figs. 3 and 4).

Besides decreasing HMGCR and lipid biosynthesis, knock-out or silencing of *Usp20* led to the increase of UCP1 and energy expenditure in BAT and iWAT (Figs. 3 and 5, and supplemenntal Fig. S3K, L, O). The effect of succinate on the thermogenic capacity of BAT suggested that succinate induced UCP1-dependent thermogenesis in BAT (36). Succinylation of UCP1 increased the protein’s stability (37). The elevated ROS from succinate dehydrogenation increased UCP1 expression by promoting differentiation to beige/brown cells from adipocyte progenitors. Their study also suggested succinate might have no effect in mature adipocytes (38). In our experiments, like L-*Usp20*^−/−^ mice, the mice that received sodium succinate had higher oxygen consumption and higher circulating succinate concentrations, and the UCP1 levels in BAT and iWAT were markedly increased in the sodium succinate-treating group (Fig. 6E–I). Compared with the control siRNA groups, silencing *Usp20* by si-*Usp20* can increase the succinate content by ∼35% (supplemental Fig. S3M, N). The protein level of UCP1 in BAT was increased (Fig. 3F) too. Thus, we concluded that the UCP1 expression is higher in BAT and iWAT of USP20 LKO mice or LNP-si*Usp20*, and succinate can increase UCP1 expression.

*Ldlr*^−/−^ mice are commonly used animal models to study dyslipidemia and atherosclerotic diseases. After being fed a high-cholesterol diet, the blood lipid levels of *Ldlr*^−/−^ mice increased significantly. Due to the lack of LDL receptors, LDL accumulates in the blood and induces atherosclerosis. Since LDL cannot be transported into tissue cells, the hepatic cholesterol synthesis pathway is not inhibited by a high cholesterol diet (6). Our study showed that compared with the control groups, the si-*Usp20* showed that USP20 was reduced to ∼30%, HMGCR was decreased to ∼50%, reduced plaque area to ∼10%, and reduced lipid levels in serum and liver. No difference was observed between control siRNA groups (Fig. 7 and supplemental Fig. S3E). The liver weight, ALT in serum, kidney weight, expression of inflammatory factors, and ER stress markers-related genes were all similar among the two groups (Fig. 4O, P, supplemental Fig. S3C, D, G, Q). Simultaneously, si-*Usp20* induced a slight increase in serum AST (supplemental Fig. S3F), indicating that long-term high-dose siRNA treatment may cause some liver damage, which should be investigated further. These results suggest that the metabolic beneficial effects of *Usp20* siRNA were on-target, and silencing *Usp20* can prevent atherosclerosis.

In summary, siRNA nanoparticle targeting *Usp20* exhibits a good therapeutic effect on metabolic diseases caused by a HFD or a high-cholesterol diet, with reduced lipid levels, decreased atherosclerosis, and increased thermogenesis and insulin sensitivity, demonstrating that siRNA targeting *Usp20* holds great promise for treating metabolic syndrome.

## Data availability

All data for this manuscript are included in the manuscript.

## Supplemental data

This article contains supplemental data.

## Conflict of interest

The authors declare that they have no conflicts of interest with the contents of this article.
