# Supplementary material for: siRNA nanoparticle targeting Usp20 lowers lipid levels and ameliorates metabolic syndrome in mice
Source: J Lipid Res. 2024 Aug 22;65(9):100626. doi: 10.1016/j.jlr.2024.100626 (PMC11418111; doi:10.1016/j.jlr.2024.100626)
Supplement: supplemental Figs. S1–S4 [file mmc1.docx]

**SUPPLEMENTAL INFORMATION:**

**siRNA nanoparticle targeting *Usp20* lowers lipid levels**

**and ameliorates metabolic syndrome in mice**

Yi Ding^1, *^, Qiu-Bing Chen^2, *^, Hui Xu^1^, Dilare Adi^3^, Yi-Wen Ding^1^, Wen-Jun Luo^1^, Wen-Zhuo Zhu^1^, Jia-Chen Xu^1^, Xiaolu Zhao^1^, Xiong-Jie Shi^1^, Jie Luo^1, 2^, Hao Yin^1,^ ^2^, Xiao-Yi Lu^1, #^

^1^ College of Life Sciences, Hubei Key Laboratory of Cell Homeostasis, Taikang Center for Life and Medical Sciences, Taikang Medical School, Wuhan University, Wuhan 430072, China

^2^ Department of Urology, Frontier Science Center for Immunology and Metabolism Medical Research Institute, Zhongnan Hospital of Wuhan University, Wuhan University, Wuhan 430072, China

^3^ Heart Center, First Affiliated Hospital of Xinjiang Medical University, Urumqi 830054, Xinjiang, China

**
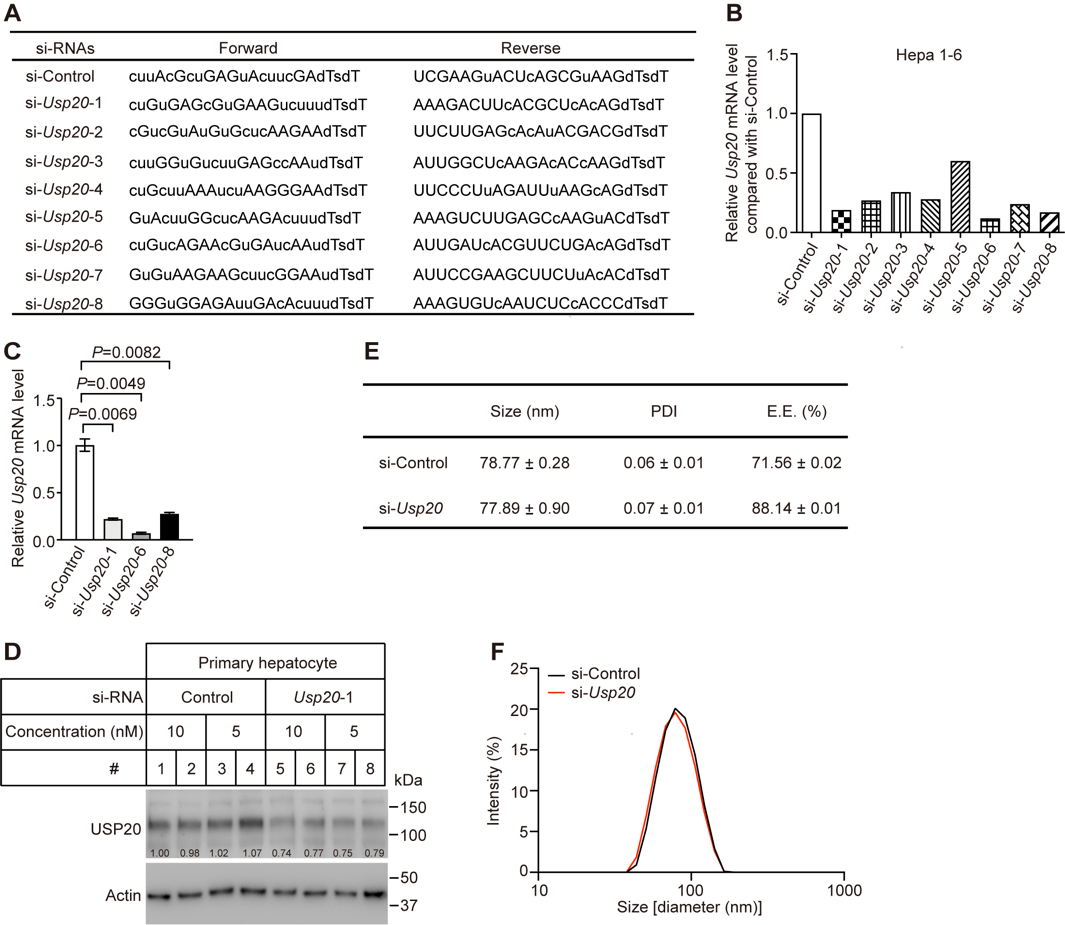
**

**Supplemental Figure S1:** **Lipid nanoparticles preparation and characterization.** (A) Sequences of the siRNAs including multiple chemical modifications. The lowercase letters c and u refer to RNA nucleotides that are modified with 2'-O-methoxyethyl (2'-OMe), while the uppercase letters A, G, C, and U refer to unmodified RNA nucleotides. dT refers to DNA thymine, and s indicates a thiol modification. (B) Screening of optimal siRNA in Hepa1-6 cells based on mRNA knockdown analyzed 24 hours post-transfection of specific siRNA. (C, D) The mRNA and protein levels analyzed 24 hours post transfection of indicated siRNAs in primary hepatocytes (C). The relative USP20 protein knockdown at the indicated concentrations in primary hepatocytes (D). (E) Physicochemical property characterization of si-Control-LNP and si-*Usp20*-LNP, which includes the size, polydispersity index (PDI), and encapsulation efficiency (E.E.). (F) Intensity-based size**
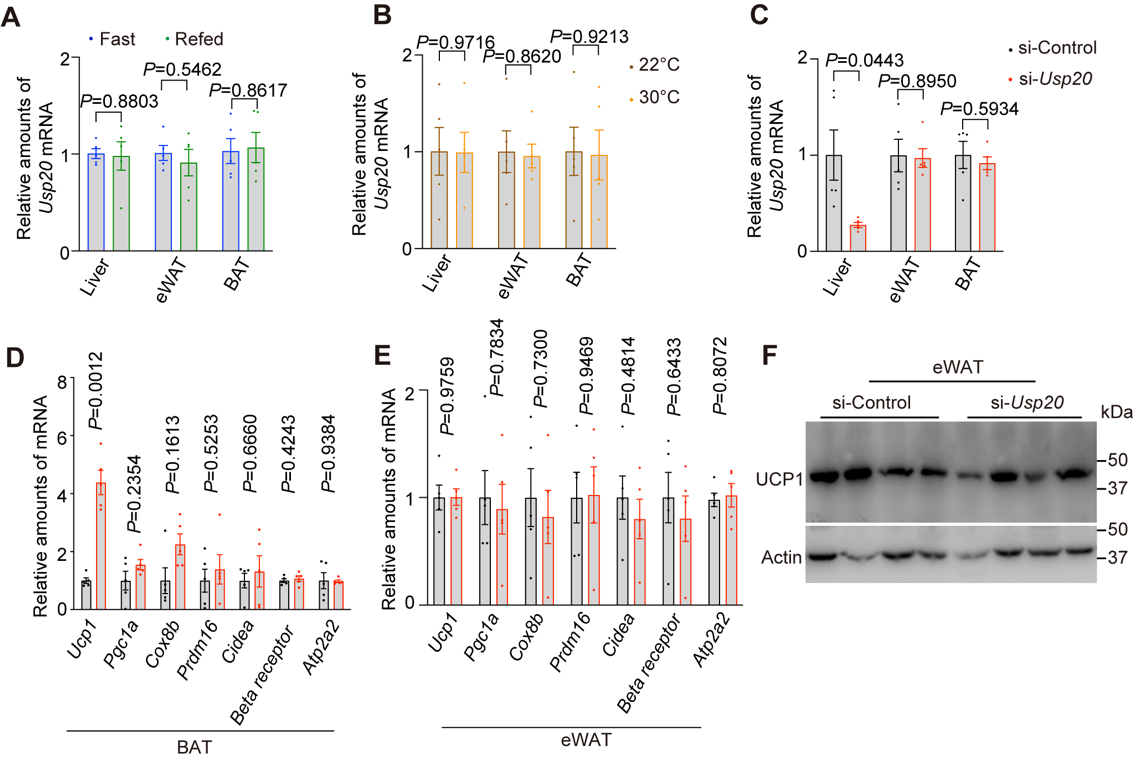
** distribution of si-Control- LNP and si-*Usp20*-LNP.

**Supplemental Figure S2: The expression of the indicated genes.**

(A) The *Usp20* mRNA level in liver, eWAT and BAT of C57BL/6 mice after fasting or refeeding treatment. (B) The *Usp20* mRNA level in liver, eWAT and BAT of C57BL/6 mice maintained at 22 ℃ or 30 ℃. (C) The *Usp20* mRNA level in liver, eWAT and BAT of mice injected with si-Control or si-*Usp20*. (D, E) The mRNA level of thermogenic gene in BAT and eWAT of mice injected with si-Control or si-*Usp20*. (F) Immunoblotting of UCP1 protein in eWAT of mice injected with si-Control or si-*Usp20*.

**
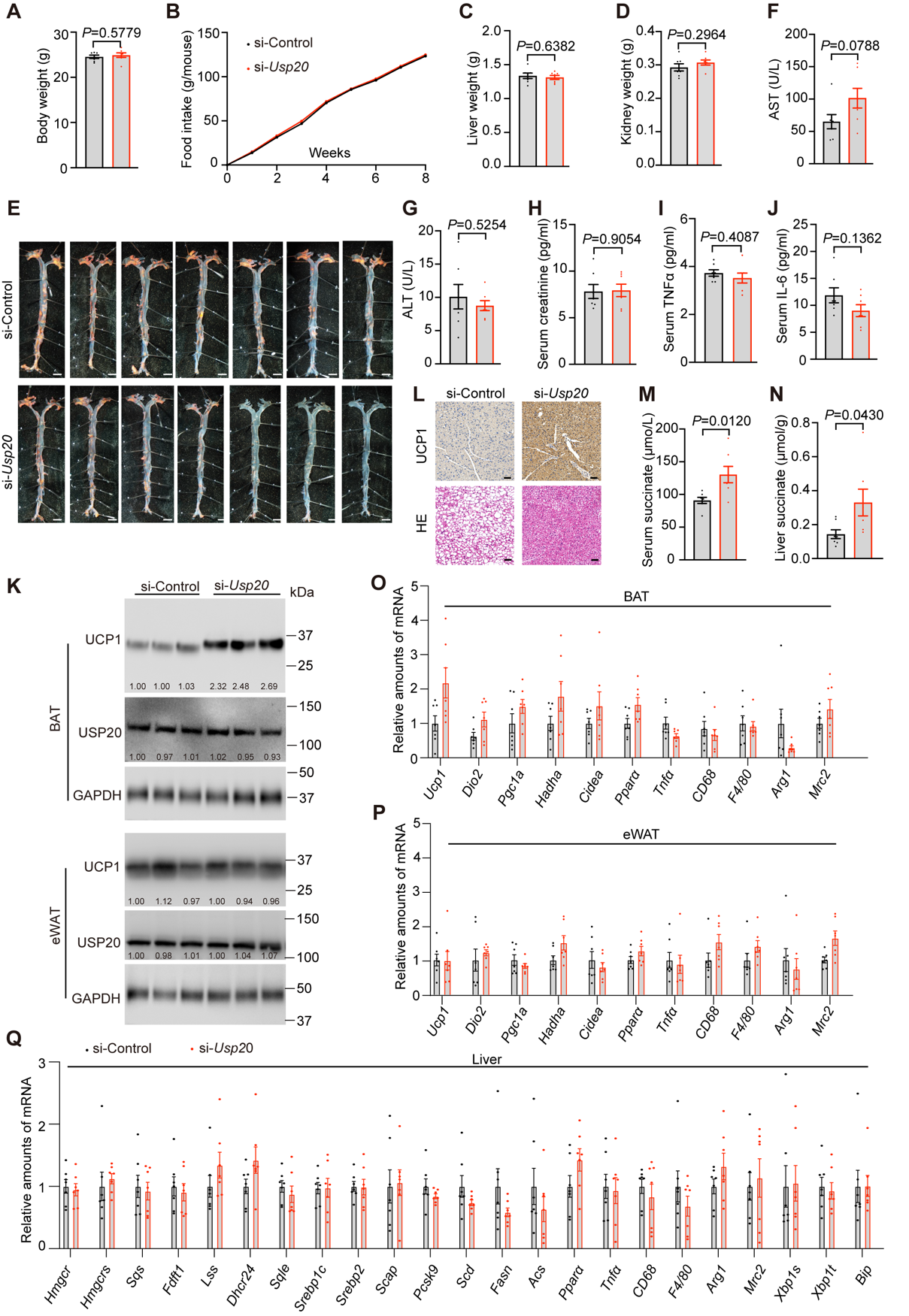
**

**Supplemental Figure S3: Characterization of the mice receiving siRNA targeting *Usp20* in the liver.**

*Ldlr*^-/-^ mice injected with different siRNA (n=7 per group) and fed high fat high cholesterol diet for 8 weeks. (A) The body weight. (B) Food intake. (C, D) The liver and kidney weight. (E) En face lipid staining of aortas of all mice (n=7 per group), of which two and Figure 7D are reused. Scale bars, 1 mm. (F-J) Serum levels of AST (F), ALT (G), creatinine (H) TNFα (I) and IL-6 (J). (K) Immunoblotting analysis of BAT and eWAT samples. (L) Representative HE and UCP1 staining of BAT sections. Scale bars, 50 μm. (M, N) Serum and liver succinate levels. (O-Q) Relative amounts of mRNA in the BAT, eWAT and liver tissues. Data are presented as mean ± SEM. *P* values were calculated by unpaired two-tailed Student’s *t-test*.

**
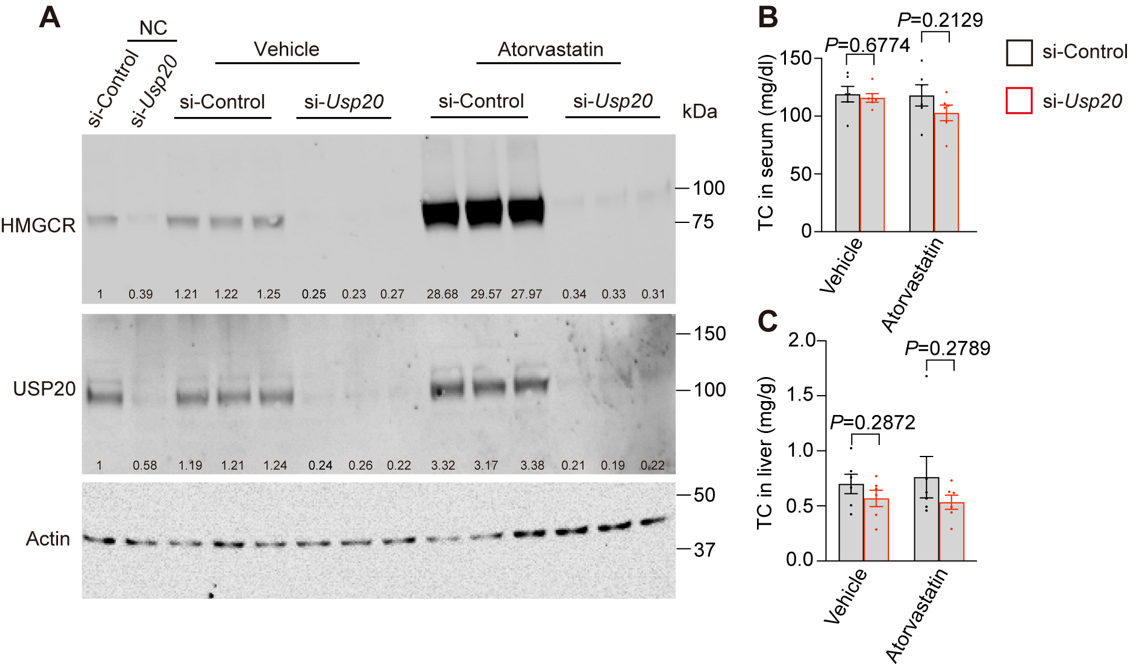
Supplemental Figure S4: Silencing *Usp20* can blunt the increase of HMGCR caused by statin.**

C57BL/6J mice were injected with 0.5 mg/kg body weight different siRNA once, and then orally gavaged with methylcellulose solution with or without 15 mg/kg/day atorvastatin for 3 consecutive days. (A) Immunoblotting analysis of hepatic HMGCR and USP20 proteins. (B, C) Total cholesterol levels in serum and liver. Data are presented as mean ± SEM. *P* values were calculated by unpaired two-tailed Student’s *t-test*.
